# The Road from AKI to CKD: Molecular Mechanisms and Therapeutic Targets of Ferroptosis

**DOI:** 10.1038/s41419-023-05969-9

**Published:** 2023-07-13

**Authors:** Runzhi Guo, Jiayu Duan, Shaokang Pan, Fei Cheng, Yingjin Qiao, Qi Feng, Dongwei Liu, Zhangsuo Liu

**Affiliations:** 1 Research Institute of Nephrology, Zhengzhou University, the First Affiliated Hospital of Zhengzhou University, Zhengzhou, 450052 P. R. China; 2 Traditional Chinese Medicine Integrated Department of Nephrology, the First Affiliated Hospital of Zhengzhou University, Zhengzhou, 450052 P. R. China; 3 Henan Province Research Center for Kidney Disease, Zhengzhou, 450052 P. R. China; 4 Key Laboratory of Precision Diagnosis and Treatment for Chronic Kidney Disease in Henan Province, Zhengzhou, 450052 P. R. China; 5 Blood Purification Center, the First Affiliated Hospital of Zhengzhou University, Zhengzhou, 450052 P. R. China

**Keywords:** Acute kidney injury, Chronic kidney disease, Cell death

## Abstract

Acute kidney injury (AKI) is a prevalent pathological condition that is characterized by a precipitous decline in renal function. In recent years, a growing body of studies have demonstrated that renal maladaptation following AKI results in chronic kidney disease (CKD). Therefore, targeting the transition of AKI to CKD displays excellent therapeutic potential. However, the mechanism of AKI to CKD is mediated by multifactor, and there is still a lack of effective treatments. Ferroptosis, a novel nonapoptotic form of cell death, is believed to have a role in the AKI to CKD progression. In this study, we retrospectively examined the history and characteristics of ferroptosis, summarized ferroptosis’s research progress in AKI and CKD, and discussed how ferroptosis participates in regulating the pathological mechanism in the progression of AKI to CKD. Furthermore, we highlighted the limitations of present research and projected the future evolution of ferroptosis. We hope this work will provide clues for further studies of ferroptosis in AKI to CKD and contribute to the study of effective therapeutic targets to prevent the progression of kidney diseases.

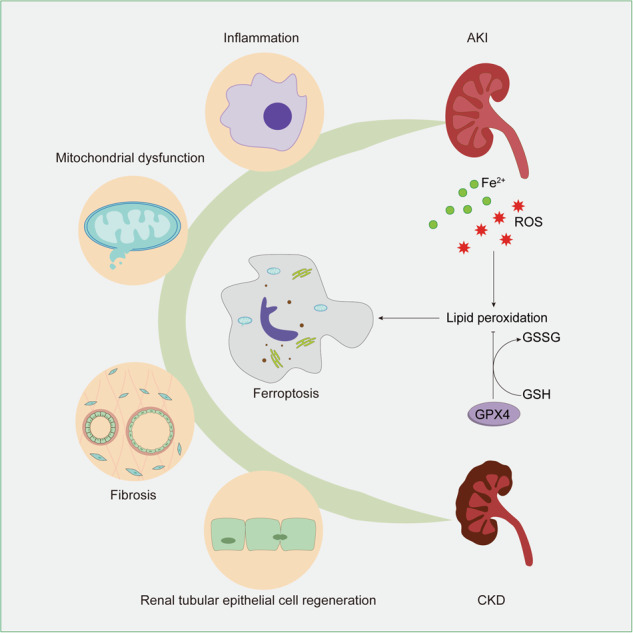

## Facts

- Ferroptosis has developed rapidly since its discovery ten years ago.
- Ferroptosis plays an important role in the transition from acute kidney injury to chronic kidney injury.
- Targeting ferroptosis provides new promising targets for preventing the progression of kidney injury.
- Studies on ferroptosis in the process of AKI to CKD are warranted and of great necessity in the future.

## Introduction

Acute kidney injury (AKI), one of the most prevalent and severe clinical diseases with high morbidity and mortality, is characterized by a sharp decline in renal filtration function. AKI is reported in 12.2% of hospitalized patients [1], with an even more significant number of people in low- and middle-income countries. AKI causes kidney tissue damage, resulting in elevated blood creatinine, increased urine protein, and decreased urine volume. The AKI-related mortality in adults is 23.9%, and the rate will be higher in those receiving kidney replacement therapy, resulting in irreversible losses to individuals and society [2].

**Fig. 1:**
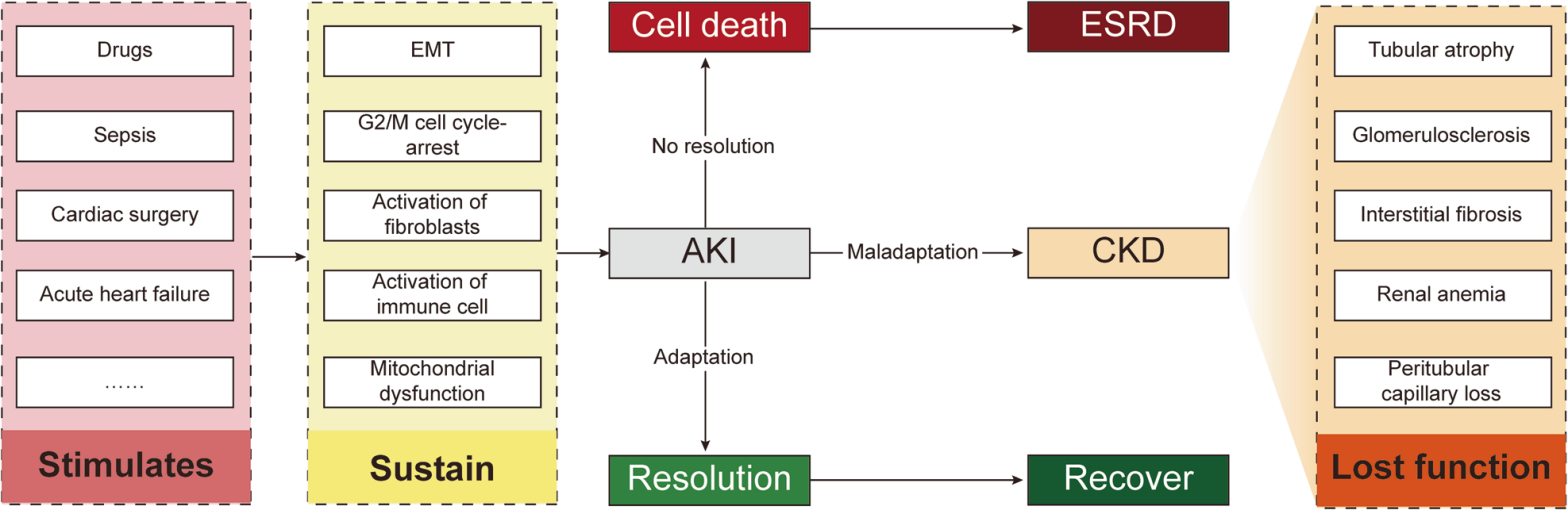
Evolution after kidney damage. Adverse stimuli such as infection, nephrotoxic drugs, and hypoperfusion induce a series of reactions, including the immune system response and cell metabolic reprogramming in the kidney. Continuous and severe stimulation can lead to cell death, organ failure, and end-stage renal disease (ESRD). Moreover, different responses to cell repair determine different prognoses. Some cells are repaired, regenerated, and cured, while maladaptive cells undergo renal tubular atrophy, renal interstitial fibrosis, and glomerulosclerosis through AKI to CKD and gradually enter the ESRD stage. EMT epithelial-mesenchymal transition, AKI acute kidney injury, CKD chronic kidney disease, ESRD end-stage renal disease.

The AKI etiology is complex and heterogeneous. Infection, sepsis, hypoxia, nephrotoxic drugs, and many other factors can cause AKI occurrence [3]. In response to nociceptive stimulation, renal cells undergo G2/M cell cycle arrest, cell senescence, and other processes by cell signal transduction and activate various forms of programmed cell death, such as ferroptosis [4]. Subsequently, AKI patients may progress to chronic kidney disease (CKD), and in some cases, die (Fig. 1). CKD, a significant global health burden, affects up to 1.2 million people annually and is projected to become the fifth leading cause of death worldwide by 2040 due to its increasing prevalence [5, 6].

Previously, it was believed that AKI and CKD were only distinguished by the duration of renal function decline. However, in the last decade, increasing evidence has suggested that AKI is an independent risk factor for CKD [7]. There is no effective treatment for AKI except for renal replacement therapy. When AKI progresses to CKD, it significantly affects patients’ survival and quality of life. To understand its pathogenesis, it is necessary to investigate the critical interval between AKI and CKD.

Recently, as a new type of cell death, ferroptosis plays a unique role in the progression from AKI to CKD, including tubular cell regeneration and interstitial fibrosis [8]. Identifying the role of ferroptosis can broaden our understanding of the pathogenesis and lead to novel prevention strategies.

## An overview of ferroptosis

**Fig. 2:**
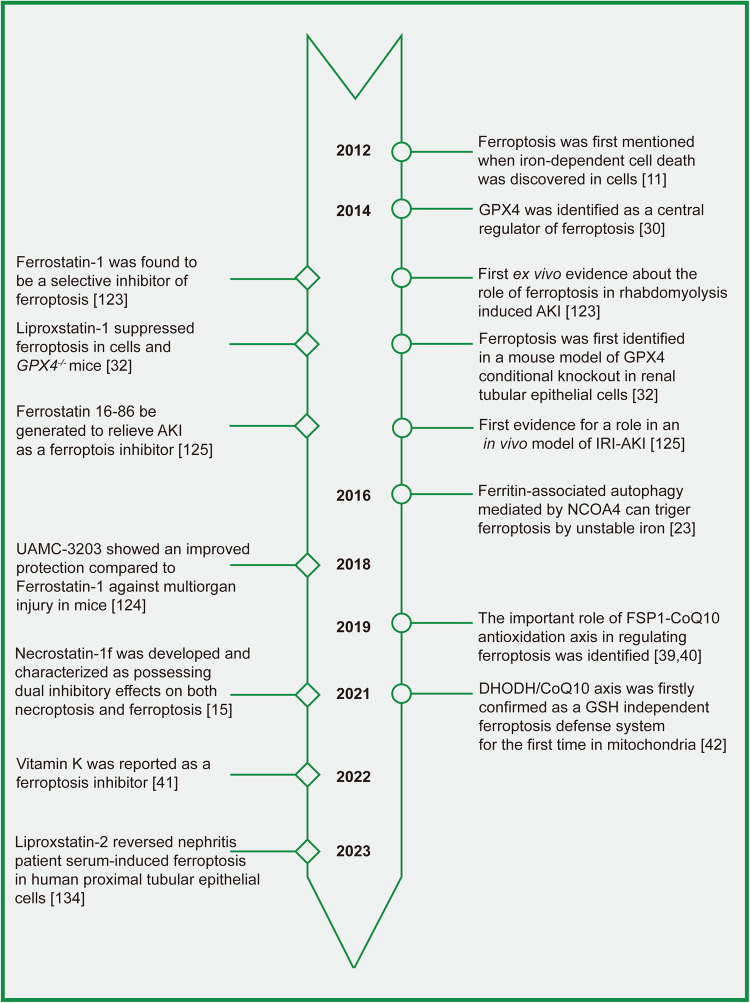
Timeline of the development of key discoveries in ferroptosis regulatory mechanisms and inhibitors. This figure depicts a timeline of significant discoveries in the regulation of ferroptosis and the creation of inhibitors for it. The timeline emphasizes crucial events, such as the initial identification of ferroptosis in cells, in animal models, and in kidney disease. The purpose of this figure is to provide a comprehensive summary of the advancements made in comprehending and treating ferroptosis from different perspectives.

Cell death is vital in organism development, homeostasis maintenance, and the occurrence and development of diseases [9]. Ferroptosis, a novel form of cell death based on iron-dependent lipid peroxidation, was first discovered in 2003 [10] and formally named in 2012 [11]. Since then, the average annual rise of ferroptosis-related studies has been a stunning 103.78%. Bibliometric analysis is a well-established method for the quantitative assessment of academic productivity. Nonetheless, the productivity of ferroptosis research has been evaluated infrequently to date. In the process of exploration in the field of ferroptosis, the regulatory mechanism of ferroptosis has been gradually discovered. Some landmark events and important ferroptosis inhibitors are shown in Fig. 2.

**Fig. 3:**
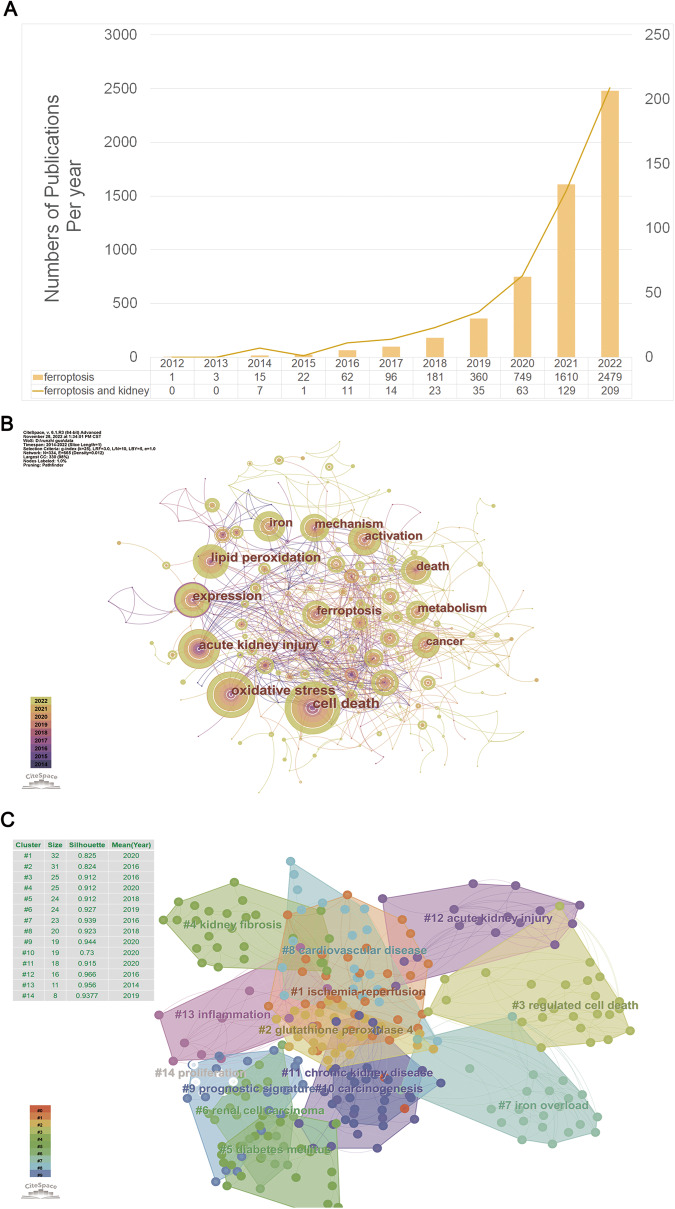
Bibliometric analyses of ferroptosis in the kidney. Data were extracted from the Web of Science database, and bibliometric analysis was performed using CiteSpace, a web-based Java application for data analysis and visualization. The keywords finally identified as follows: 1st: [TS = ferroptosis or TS = ferroptotic) and ((PY = (2012–2022)) AND DT = (Article OR Review)) AND LA = (English). 2nd: [TS = ferroptosis or TS = ferroptotic) and (((((TS = (kidney)) OR TS = (renal)) OR TS = (nephr*)) OR TS = (Glomer*)) OR TS = (podocyte)) OR TS = (“Proximal tubular”) and ((PY= (2012–2022)) AND DT = (Article OR Review)) AND LA = (English) (**A**) Number of publications per year; (**B**) Keywords co-occurrence analysis; (**C**) Keywords cluster view.

Figure 3 displays the findings of our bibliometric analysis of the research publications published on ferroptosis in the decade following its formal designation in 2012.Based on the Web of Science (www.isiknowledge.com/), we analyzed the scientific output related to ferroptosis from 2012 to 2022. In total, 5578 articles relating to hypertension were identified in the Web of Science. To further explore the research progress of ferroptosis in the field of kidney diseases, we further added kidney-related keywords, and the number of retrieved articles was 492.

We analyzed the trend of ferroptosis and ferroptosis-related research in the kidney using histograms and line graphs (Fig. 3A), showing the same upward trend since its official naming in 2012, particularly in 2019–2022. High-frequency keywords represent the hot spots in a research field and reflect the status and influence of the corresponding study content in the research field. Through the analysis of high-frequency keywords in the literature (Fig. 3B), we found that the top keywords in the order of frequency and centrality were cell death (frequency: 129, centrality: 0.02), oxidative stress (frequency: 115, centrality: 0.11) and acute kidney injury (frequency: 81, centrality: 0.17). Other keywords included lipid peroxidation, iron, mechanism, and metabolism. A cluster analysis of cooccurring keywords revealed the main themes using CiteSpace (Fig. 3C). The modularity of our analysis is 0.7428, and the mean silhouette is 0.8802, supporting that our clustering results are credible. The clustering results showed that studies on ferroptosis in kidney diseases focused on AKI, CKD, and ischemia‒reperfusion injury (IRI). Meanwhile, the relevant research progress is mainly linked with the regulatory roles of ferroptosis in kidney injury, renal fibrosis, inflammation, and other mechanisms.

## Ferroptosis and other types of cell death

As a recently discovered form of cell death, ferroptosis possesses distinctive morphological and biochemical properties that distinguished it from apoptosis, autophagy, and necroptosis (Table 1). Typical morphological changes of ferroptosis were revealed by transmission electron microscope, which mainly showed shrunken and damaged mitochondria with thickened membranes and a reduction or loss of mitochondrial cristae [12]. The biochemical characteristic of ferroptosis is intracellular iron accumulation and excessive reactive oxygen species (ROS) burst, leading to lipid peroxidation and mitochondrial dysfunction.

**Table 1:** Main types of programmed cell death [135–137].

	Ferroptosis	Necroptosis	Apoptosis	Autophagy	Cuproptosis	Pyroptosis
Morphology	Mitochondrial volume decreased; membrane density increased with the mitochondrial crest decreased or disappeared	Cellular swelling; cell membrane rupture; cell membrane forms selective ion channels	Cell shrinkage; nuclear volume decrease; plasma membrane blebbing with apoptotic bodies	Formation of double-membrane autophagosome and autophagic vacuoles; nuclear fragmentation with amorphous cytoplasm; swelling of Golgi apparatus and endoplasmic reticulum	Mitochondrial shrinkage; mitochondrial membrane rupture	Cellular swelling; cell membrane forms nonselective channels; plasma membrane blebbing with pyroptotic bodies
Key regulators	GPX4, ACSL4, NCOA4, SLC7A11	RIPK1, RIPK3, MLKL	Caspase-3, Fas, Bcl-2, Bax	ATG5, ATG7, LC3	DLAT, FDX1, LIAS, CTR1, ATP7B	Caspases, Gasdermin D, IL-1β, IL-18
Biochemical characteristics	Lipid peroxide accumulation and iron overload	Inflammatory responses	DNA degradation	High level activity of lysosomes	Copper accumulation; copper-bound lipid protein aggregation; protein lipoylation	Cell converts into pore-induced intracellular traps

In addition, the crosstalk between ferroptosis and other forms of cell death has been studied in recent years. A wave of noncell-autonomous kidney tubular injury occurs during AKI, and Belavgeni et al. suggested that necroptosis may initiate the spread of cell death via ferroptosis [13]. Necroptosis to ferroptosis may be achieved through phosphatidylethanolamine-binding protein 1 and 15-lipoxygenase [14]. Based on the interconnected relationship between necroptosis and ferroptosis, a combined small molecule inhibitor Necrostatin-1f was created, which has a strong inhibitory effect on necroptosis and a weak inhibitory effect on ferroptosis [15]. There may also be a relationship between pyroptosis and ferroptosis. Iron ions and ROS-induced drugs induce pyroptosis through the ROS-Tom20-Caspase3-GSDME signaling pathway [16]. There have been studies on the simultaneous targeting of ferroptosis and pyroptosis for tumor therapy [17], but there is a lack of more evidence on the crosstalk between pyroptosis and ferroptosis in kidney disease models. We look forward to related studies in the future to fill this gap.

## Key metabolic mechanisms of ferroptosis

**Fig. 4:**
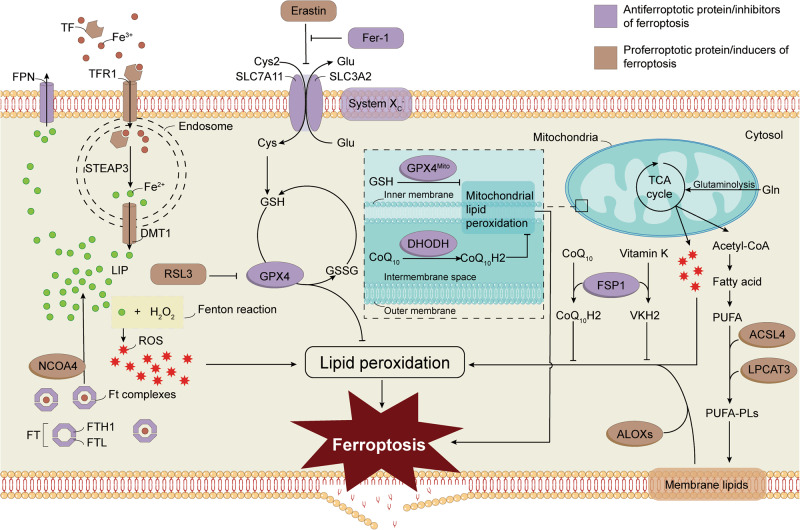
Schematic diagram of the primary regulatory mechanisms associated with ferroptosis. Lipid peroxidation is necessary for ferroptosis in individual cells, in which iron-induced ROS burst and the decrease of antioxidation are required. Several molecular mechanisms were reported to regulate the occurrence of ferroptosis, such as the system Xc^-^/GPX4 antioxidant axis, iron regulons NCOA4 and DMT1. Furthermore, ACSL4, LPCAT3, and ALOXs induced ferroptosis by influencing the levels of cellular lipid peroxides. ROS reactive oxygen species, Cys2 cystine, Cys cysteine, Glu glutamate, GSH glutathione, Gln glutamine, TCA cycle tricarboxylic acid cycle, DHODH dihydroorotate dehydrogenase, CoQ coenzyme Q, GPX4 glutathione peroxidase 4, PUFAs polyunsaturated fatty acids, ACSL4 acyl-CoA synthetase long-chain family member 4, LPCAT3 lysophosphatidylcholine acyltransferase 3, FSP1, ferroptosis suppressor protein 1, ALOX arachidonate 5-lipoxygenase, TF transferrin, TFR1 transferrin receptor 1, DMT1 divalent metal transporter 1, Ft ferritin, FTH1 ferritin heavy chain, FTL ferritin light chain, NCOA4 nuclear receptor coactivator 4, Fer-1 ferrostatin-1, FPN ferroportin, LIP labile iron pool, SLC7A11 solute carrier family 7 member 11, SLC3A2 solute carrier family 3 member 2, STEAP3 six-transmembrane epithelial antigen of the prostate 3.

The direct factor leading to ferroptosis is lipid peroxidation, which is regulated by iron metabolism, system Xc^-^, antioxidant molecules, and polyunsaturated fatty acids (PUFAs) generation (Fig. 4). Herein, we systemically elaborate on the regulatory mechanisms of ferroptosis in the following aspects.

### Iron metabolism

Iron overload is a prerequisite for ferroptosis, and iron metabolism is crucial in ferroptosis regulation. Iron is an essential element in the human body and participates in many physiological activities [18]. Iron homeostasis in cells is exquisitely regulated. Iron ions in the food are absorbed by mesenteric cells in the form of Fe^2+^ and then transformed into Fe^3+^ by ceruloplasmin. Fe^3+^ binds to transferrin and is subsequently absorbed into renal cells via transferrin receptor 1 [19]. In the endosome, Fe^3+^ is reduced to Fe^2+^ by the six-transmembrane epithelial antigen of prostate 3, which then transport to cytoplasm by divalent metal transporter 1 at the membrane [20]. Most excess iron is stored by ferritin in its inactive form, and a small fraction of Fe^2+^ forms a labile iron pool (LIP) [21]. Ferritin (Ft) is composed of FTH and FTL. The Fe^2+^-Ft complex can be targeted by nuclear receptor coactivator 4 to release iron by autolysosomal degradation, which triggers ferritinophagy and ultimately increases cell susceptibility to ferroptosis [22, 23]. Intracellular Fe^2+^ can react with hydrogen peroxide and produce a large number of hydroxyl radicals with strong oxidation by the Fenton reaction [24]. Under pathological conditions, the accumulation of Fe^2+^ leads to excessive ROS production and mediates ferroptosis [25].

### Amino acid metabolism

The system Xc^-^-GPX4 axis is the earliest and most important regulator in suppressing ferroptosis and is crucial in antioxidant system. System Xc^-^, also named cystine/glutamate antiporter, is a transmembrane transport complex that is composed of a catalytic subunit solute carrier family 7 member 11 (SLC7A11) and a regulatory subunit solute carrier family 3 member 2 (SLC3A2) [26, 27], which can transfer glutamate output to the cell and cysteine to the cell in a ratio of 1:1 [27]. Cysteine is the rate-limiting precursor for the biosynthesis of reduced glutathione (GSH) [28]. GSH is composed of glutamate, cysteine, and glycine, and it can remove membrane lipid peroxides under the synergistic action of glutathione peroxidase 4 (GPX4) [29]. GPX4 catalysts the reduction process and detoxifies lipid ROS production [30]. Phospholipid hydroperoxides (PLOOH) are an oxidative product of PUFAs and an activator of the peroxidation chain reaction [31]. GPX4 has PLOOH-neutralizing enzyme activity and can reduce PLOOHs to PLOHs with the assistance of GSH [31]. The antioxidant effects of the system Xc^-^-GPX4 axis are mainly manifested in maintaining cell homeostasis, reducing oxidative stress, and inhibiting ferroptosis. Erastin is a classical ferroptosis activator, which can inhibit activity of system Xc^-^ [10], thereby resulting in GSH exhaust, lipid peroxide accumulation, and eventually causing cell death. GPX4 is also a star molecule in ferroptosis regulation that can effectively inhibit the occurrence of ferroptosis and has been considered a promising therapeutic target in many diseases [29]. RSL3 is another ferroptosis activator, which can bind to and inactivate GPX4 [30]. Knockout of GPX4 in mice induces AKI [32]. Several recent studies have proven that p53 regulates ferroptosis through system Xc^-^ and GPX4 [33].

In addition to being regulated by the system Xc-, GSH is also regulated by dipeptidase-1(DPEP-1). DPEP-1 is prominently expressed on proximal tubular epithelial cell (PTEC) and peritubular capillaries of the kidney, where it functions as a significant adhesion receptor for neutrophils. Previous studies have found that DPEP-1 can assist in the renal tubular reabsorption of contrast agents that exacerbate cisplatin-induced AKI, and regulate the adhesion of neutrophils and monocytes to peritubular capillaries during kidney IRI, thus playing an important role in the inflammatory response of IRI-AKI [34, 35]. In addition, DPEP-1 can degrade GSH and participates in the regulation of oxidative stress [36]. Interestingly, in 2022, von Mässenhausen *et al*. found that dexamethasone decreased GSH expression by upregulating DPEP-1, thereby increasing the sensitivity of PTEC to ferroptosis [37]. However, the current evidence supports the regulatory effect of DPEP-1 on ferroptosis is far less than that of system Xc-, and DPEP-1 inhibitors or knockout of DPEP-1 cannot reverse the erastin-induced ferroptosis. Whether the overexpression of DPEP-1 can induce ferroptosis or not, and the comparison of DPEP-1 with other ferroptotic triggers needs to be further studied.

In addition to the system Xc^-^/GPX4 antioxidant axis, recent studies have found that GPX4-independent systems, such as the ferroptosis suppressor protein 1 (FSP1)/coenzyme Q_10_ (CoQ_10_) axis and the dihydroorotate dehydrogenase (DHODH)/CoQ_10_ axis [38]. The FSP1/CoQ_10_ axis was discovered in 2019 [39, 40]. Accumulating evidence suggests that FSP1 is likely to be the second pillar of ferroptosis regulation after GPX4. The antioxidant effect of FSP1 is achieved by reducing CoQ_10_ to CoQ_10_H2, which produces lipophilic free radicals that capture antioxidants and decrease lipid peroxide accumulation in tissue. In recent years, it has demonstrated that FSP1 can catalyze the reduction of vitamin K to produce VKH2 to capture free radicals and prevent lipid peroxidation and ferroptosis [41]. DHODH, a mitochondrial inner membrane enzyme that catalyzes the synthesis of de novo pyrimidine ribonucleotides, was recently found to act as an antioxidant and target ferroptosis by reducing CoQ_10_ in a manner independent of mitochondrial GPX4 [42].

### Lipid metabolism

The lethal accumulation of lipid ROS is a vital link in ferroptosis. There are many different classes of lipids in cells, including fatty acids, phospholipids, and cholesterol, among which PUFAs are more sensitive to lipid peroxidation [43]. PUFAs on the lipid membrane react with ROS and are then oxidized, driving the occurrence of ferroptosis [44]. Studies have shown that phosphatidylethanolamine (PE), a PUFA-related phospholipid that contains arachidonic acid (AA) or its derivative adrenaline, is the essential phospholipid that induces cell ferroptosis [45].

Acyl-CoA synthetase long-chain family member 4 (ACSL4) and lysophosphatidylcholine acyltransferase 3 (LPCAT3) play essential roles in the PUFAs metabolism regulation. Free PUFAs must be esterified to membrane phospholipids and oxidized to transmit ferroptosis signals, and ACSL4 and LPCAT3 can regulate the conversion of free PUFAs to membrane phospholipids [46]. Knockdown or inhibition of ACSL4 can alleviate ferroptosis-induced tissue injury after IRI by reducing lipid peroxidation [47].

Lipoxygenases (LOXs) are a class of dioxygenases that can directly oxidize PUFAs and PUFA-containing lipids in biofilms [43] and are also promising regulators of ferroptosis. Ferroptosis was inhibited by LOX inhibitors or siRNA-mediated silencing of *ALOX15*, suggesting a link between LOXs and ferroptosis [48]. However, the *ALOX15* knockout in GPX4 conditional knockout mice did not prevent ferroptosis [32]. Therefore, the precise role of LOXs in ferroptosis remains unknown and requires further study.

## The role of ferroptosis in the pathogenesis of AKI to CKD progression

Cellular noxious stimulation triggers harmful molecule release, mitochondrial damage, immunocyte and fibroblast activation. The body activates protective mechanisms, promoting cellular repair and regeneration.The process from AKI to CKD is essentially a process of cellular maladaptation [49]. Once the disease enters the chronic phase, restoring damaged tissues to normal is difficult, which is associated with organ dysfunction, high morbidity and high mortality. A potential strategy to overcome this challenge is targeting common mechanisms and core pathways with central pathophysiological relevance in different pathological alterations in AKI to CKD [50]. Ferroptosis plays a vital role in regulating various cellular processes, such as inflammation [51], mitochondrial dysfunction [52], fibrosis [53], and renal cell regeneration [54], and has been considered a promising therapeutic target in the progression of AKI to CKD.

### Inflammation

**Fig. 5:**
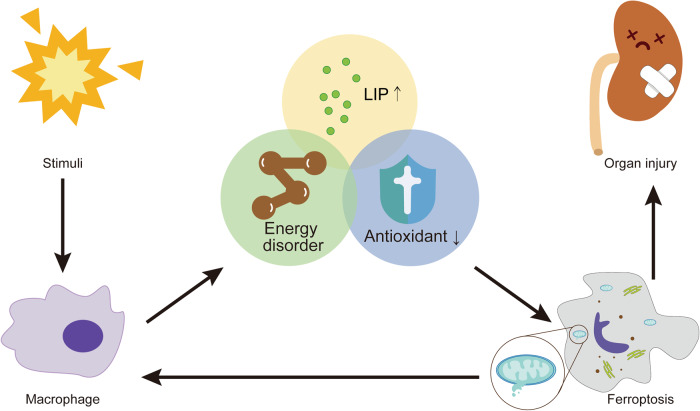
The crosstalk between inflammation and ferroptosis. As a major representative cell of the inflammatory response, macrophages are activated in response to PAMPs and DAMPs after intense noxious stimulation. The inflammatory state may have led to an energy disorder, increased the level of LIP, and attenuated the beneficial effect of antioxidants, which further triggered ferroptosis. Cell death leads to the release of DAMPs which stimulate inflammation. Thus a positive feedback loop forms and ultimately leads to organ injury.

Inflammation is a complex biological response of the body to pathogens or tissue damage. The pattern recognition receptors (PRRs) of immune systemcan recognize pathogen-associated molecular patterns (PAMPs) to defend against infection and tissue damage [55]. In addition, the immune system can respond to intracellular damage by sensing endogenous stimuli called danger-associated molecular patterns (DAMPs) [56]. After AKI, the inflammatory cascade occurs in the acute phase; however, persistent chronic inflammation stimulation promotes AKI to CKD [57]. This section discusses the crosstalk between inflammation and ferroptosis in AKI to CKD (Fig. 5).

#### Ferroptosis can be exacerbated by inflammation

Although AKI is caused by numerous pathogenic factors, cell damage can induce inflammatory cascades that lead to cell death. In the initial stage of injury, some DAMPs (e.g., heat shock proteins and histones) are released to the outside of the cell, which causes tissue-resident cells such as dendritic cells and fibroblasts to secrete proinflammatory cytokines and chemokines by activating PRRs, thus causing cell death [58]. Therefore, ferroptosis is closely associated with the inflammatory state, which may be related to metabolic disorders of iron, lipid and energy in the organism during severe infection [59]. Increased levels of LIP caused by increased iron transport and decreased iron export during sepsis can lead to ferroptosis [60]. Moreover, the inflammatory state is related to the production of high-energy metabolites such as lactate and free fatty acids, which may also be a key factor for the vulnerability of the kidney to ferroptosis in the inflammatory state [61]. Recent studies showed that the inflammatory state of PTEC after injury could aggravate ferroptosis and damage cells by downregulating glutathione metabolism genes [62].

#### Ferroptosis promotes an inflammatory response

Ferroptosis is highly immunogenic and can induce the release of inflammatory mediators and DAMPs [63]. The recognition of immune system to DAMPs leads to a persistent immune response and inflammatory state, thereby inducing the progress of the disease. High-mobility group box 1 (HMGB1) is a released DAMP in ferroptosis that mediates the inflammatory response activation, but it can be blocked by ferroptosis inhibitors [64]. However, how DAMPs are released during ferroptosis is unclear. ACSL4-dependent lipid biosynthesis is essential in ferroptosis, and its expression positively correlates with ferroptosis process [65]. Meanwhile ACSL4 is also involved in inflammation, and its expression positively correlates with the abundance of immunocytes, such as macrophages, dendritic cells, neutrophils [66]. The transcription factor nuclear factor-κB (NF-κB) can be activated by the proinflammatory cytokine tumor necrosis factor α (TNF-α) and plays a crucial role in immunomodulation [67]. High intracellular ROS levels are involved in lipid peroxidation during ferroptosis and can activate the NF-κB signaling pathway [68, 69]. In mammals, GPX4 can prevent TNF-α-mediated NF-κB signaling pathway activation and attenuate necrotizing inflammation [70]. PUFAs and their metabolic enzymes are crucial in ferroptosis as substrates of lipid peroxidation. Arachidonic acid induces the inflammatory cascade as a prerequisite for proinflammatory mediators when cells are exposed to stimuli, which proves that PUFAs also play an essential role in inflammation [71–73].

In summary, when renal tissues or cells are subjected to harmful stimuli, many endogenous DAMPs and proinflammatory mediators are released, stimulating immunocytes to trigger inflammatory cascades [74–76]. Activated immunocytes release cytokines such as IL-6 and TNF-α, thus promoting the migration of antigen-presenting cells (APCs). Continuous high levels of inflammatory infiltration will accelerate cell death and organ damage [77, 78]. The dead cells release DAMPs to further aggravate the progress of tissue damage. However, a comprehensive map of the DAMPs released during ferroptosis has yet to be compiled.

Moreover, the precise processes through which ferroptosis regulates necrotizing inflammation remain unknown. Does the ferroptosis inhibitor alleviate inflammation by reducing ferroptosis, or can it directly act on inflammatory signaling pathways? This question is still unknown. Therefore, the crosstalk between ferroptosis and necrotizing inflammation needs further exploration.

### Mitochondrial dysfunction

Under physiological conditions, fatty acid metabolism and ATP production in mitochondria are essential for maintaining normal kidney function. However, mitochondrial dysfunction after kidney injury aggravates pathological changes [79]. Currently, mitochondrial dysfunction is considered to be an essential factor in the progression of AKI to CKD. Mitochondrial damage occurs in the early stage of AKI which leads to ATP deficiency, excessive ROS production, and loss of renal function [80].

Mitochondria play a central role in ferroptosis [81]. Mitochondrial morphological changes are the distinctive morphological hallmarks of ferroptosis, and mitochondria are engaged in crucial processes such as lipid and energy metabolism [52, 82]. Mitochondria can utilize iron to synthesize iron-sulfur clusters or heme prosthetic groups and then regulate their distribution and utilization in cells, which play a central role in iron homeostasis [83, 84]. Excessive iron leads to oxidative stress and mitochondrial dynamics disorder, mainly manifested as increased ROS production, decreased ATP produce, and increased glycolysis to replenish the reduced ATP, all of which promote ferroptosis [52, 85]. The disorder of fatty acid metabolism in mitochondria will lead to lipotoxicity and cell stress after AKI, which is prone to progress to CKD [86]. Furthermore, necrotic cells release mitochondria that can act as DAMPs to affect neighboring cells [87], which has been implicated in the inflammatory response in kidney injury. The most significant morphological changes in ferroptotic cells under electron microscopy were morphological changes in mitochondria [12, 88].

Mitochondrial ferritin (FtMt) is an iron storage protein in mitochondria. Overexpression of FtMt can inhibit erastin-induced ferroptosis by increasing iron storage and reducing LIP [89, 90]. Improving mitochondrial homeostasis and restoring FAO can reduce renal injury after IRI [91]. Additionally, there exists a set of antioxidant systems in mitochondria independent of the cytoplasm antioxidant systems. DHODH and mitochondrial GPX4 are the two central defense systems for mitochondrial lipid peroxide elimination [42]. Loss of one system forces the cell to become more dependent on the other, while loss of both protective systems induces ferroptosis, mainly induced by mitochondrial lipid peroxidation.

Therefore, mitochondrial dysfunction leads to a weakened role in defending against iron death, increased LIP levels and induced mitochondrial lipid peroxidation. This suggests mitochondria may be a promising therapeutic target for reducing ferroptosis and improving patient outcomes.

### Fibrosis

Renal tubulointerstitial fibrosis is a feature of incomplete epithelial repair and a significant factor in AKI and CKD. Fibrosis is an overaccumulation of the extracellular matrix, which can respond to chronic injuries in various organs, such as the liver, kidney, and heart, and is associated with poor response to treatment [92]. Thus, there is an urgent need to understand the mechanisms of fibrosis and develop new therapeutic strategies. Recent in-depth studies on ferroptosis have revealed a growing body of evidence that highlights the crucial role of ferroptosis in the pathophysiological process of fibrosis [93].

Ferroptosis and fibrosis share common metabolic pathways. In the process of fibrosis, there is metabolic reprogramming of fibroblasts caused by increased glycolysis, excessive breakdown of glutamine, and enhanced fatty acid oxidation (FAO) [94], which is also related to ferroptosis. During amino acid starvation, the increase in the glutamine-based tricarboxylic acid (TCA) cycle triggers ferroptosis [95]. Similarly, AKI leads to increased glycolysis and FAO, which are also associated with ferroptosis [85]. Ferroptosis and fibrosis have the same pathological mechanisms [96]. A decrease in FTH can be observed in epithelial-mesenchymal transition (EMT), which is an essential link in interstitial fibrosis. Then, ferritin releases free iron ions, and the increase in LIP is closely related to the rise in ROS levels and the occurrence of ferroptosis [97]. Elevated levels of LIP and ROS not only arise from fibrosis, but also play a role as mediators in regulating fibrosis occurrence. When exogenous supplementation of FTH was reduced during EMT, the degree of fibrosis was reduced [97]. ROS production due to the profibrotic cytokine transforming growth factor beta (TGF-β) leads to redox imbalance and mediates the fibrotic effects of TGF-β [98, 99].

This common pathological mechanism is well-represented in kidney diseases. In a folic acid-induced animal model of AKI, ferroptosis has been implicated in the development of renal fibrosis [100, 101]. Moreover, recent studies have shown that ferroptosis activation can promote fibrosis, and ferroptosis is also accompanied with the fibrotic process, thus forming a vicious cycle [102]. During ferroptosis, human kidney-2 (HK-2) cells secrete various profibrotic factors. When HK-2 cells were cultured with ferroptosis activator RSL3 and subsequently co-incubated with renal fibroblasts, the fibroblasts could be activated and proliferated, whereas ferroptosis inhibitor liproxstatin-1 attenuated the profibrotic effects [103]. The same results were observed in mice with IRI [104].

Current research proves that ferroptosis is closely linked to renal fibrosis with shared metabolic pathways and pathological mechanisms. Ferroptosis inhibitors can prevent or delay renal fibrosis and mediate interstitial fibroblasts’ fibrotic response. Ferroptosis can occur and mediate further aggravation of fibrosis during the progression of the renal fibrosis model. Therefore, during the progression from AKI to CKD, the risk factors causing AKI sequentially activate the ferroptosis and fibrosis pathways, and their interaction further aggravates kidney injury. Early prevention of ferroptosis can reduce renal maladaptation and delay/rescue fibrosis.

### Renal tubular epithelial cell regeneration

The proximal tubule, a potential determinant of the risk and outcomes of kidney diseases [105], is a primary site for both ferroptosis and AKI. The proximal tubule is responsible for the vital function of material transport and the reabsorption [106, 107]. Therefore, PTEC have the highest energy demand and mainly relies on fatty acids as an energy source. PTEC do not undergo glycolysis under physiological conditions; however, kidney injury can lead to metabolic disorders, and glycolysis is increased shortly after injury to compensate for energy loss [108]. The timely supply of energy can protect the kidney to some extent, but the long-term effect is not optimistic. Glycolysis in PTEC can inhibit the proliferation and differentiation of podocytes and aggravate renal interstitial fibrosis [109].

Moreover, the proximal tubules are rich in mitochondria, which are required for the oxidation of fatty acids to produce ATP. Renal tubules are damaged during AKI, leading to mitochondrial dysfunction and high levels of mitochondrial reactive oxygen species (mtROS), which contribute to ferroptosis [110, 111]. Moreover, PTEC injury is related to their dedifferentiation and cell cycle arrest, causing interstitial fibrosis and glomerular lesions.

The intrinsic repair ability of the proximal tubule after noxious stimulation makes it less damaged. After damage, PTEC dedifferentiate and proliferate to restore the nephron [112]. A clinical study showed that approximately 12.6% of patients with severe AKI recovered normal renal function after renal replacement therapy [113]. However, it is not clear what determines the outcome in different patients. Previous studies have confirmed that ferroptosis activation impairs skeletal muscle regeneration [114], affects bone remodeling in osteoporosis [115] and prevents wound healing in diabetic ulcers [116, 117]. GPX4 is involved in wound repair of the corneal epithelium [118]. Ferroptosis inhibitors can repair spinal cord and facial nerve injury and promote wound healing in diabetic patients [119–121].

However, studies on the role of ferroptosis in renal repair in AKI and CKD are still rare. Recent genetic and single-cell transcriptome analysis in mice revealed that ferroptosis determined cell plasticity and that the Nuclear factor erythroid 2-related factor 2 (Nrf2)-mediated antioxidant system protected against renal repair failure following AKI by controlling ferroptosis [122]. This study suggests that targeting ferroptosis may be the key to explaining the poor prognosis of AKI patients, but further studies are still needed.

## The potential of ferroptosis-related drugs in the treatment of AKI to CKD

As mentioned above, many small molecular compounds are known to act on ferroptosis-related molecules and thus exert nephroprotective effects. In 2014, Skouta et al. first verified the role of ferrostatin-1 in inhibiting ferroptosis in rhabdomyolysis-induced AKI model [123]. In addition, new ferroptosis inhibitor UAMC-3203 has been verified to possess better therapeutic potential than ferrostatin-1, but it has not been evaluated in AKI and CKD models [124]. In this section, we will summarize and elaborate on the current research progress of ferroptosis-related drugs in treating AKI to CKD (Table 2).

**Table 2:** Ferroptosis-related drugs are involved in the treatment of AKI to CKD.

Drugs	Animal models	Mice types	Cell types	Mechanisms	References
Liproxstatin-1	UUO	C57BL/6 mice	TEC, HK-2	Reduce lipid peroxidation	[103]
Ferrostatin-1	Cis-AKI	CD1 mice	HK-2	Reduce lipid peroxidation	[138]
Ferrostatin 16-86	IRI-AKI	C57BL/6 mice	Primary mouse renal tubules	Reduce lipid peroxidation	[125]
Vitamin E	IRI-AKI	C57BL/6 mice	RPTECs	Antioxidant	[139]
Vitamin K	IRI-AKI	C57BL/6 mice	RPTECs	Antioxidant	[41]
Paricalcitol	Cis-AKI	C57BL/6 mice	HK-2	Antioxidant	[131]
Irisin	IRI-AKI	C57BL/6 J mice	HK-2	Antioxidant	[140]
Tectorigenin	UUO	C57BL/6 mice	RPTECs	Antioxidant	[128]
Isoliquirtigenin	LPS-AKI	C57BL/6 mice	HK-2	Antioxidant	[141]
Melatonin	IRI-AKI, FA-AKI	C57BL/6 J mice	TEC	Antioxidant	[142]
Quercetin	IRI-AKI, FA-AKI	C57BL/6 J mice	NRK-52E, HK-2	Antioxidant	[143]
Deferoxamine	CKD	Sprague‒Dawley rat	–	Iron chelator	[132]
Nobiletin	UUO	C57BL/6 J mice	–	Antioxidant; anti-inflammatory	[129]

*UUO* unilateral ureteral obstruction, *Cis-AKI* cisplatin-induced acute kidney injury, *RPTECs* primary renal proximal tubular epithelial cells, *MH-ARF* myohemoglobinuric acute renal failure.

Inhibition of ferroptosis alleviates the inflammationafter renal tissue injury, and this improvement may be attributed to the decreased release of DAMPs. In conclusion, animals with AKI produced by ischemia‒reperfusion or oxalate crystals exhibit decreased leukocyte migration when treated with ferroptosis inhibitor ferrostatin 16-86 [125]. Furthermore, inhibiting PTEC ferroptosis reduced monocyte chemoattractant protein-1 (MCP-1) secretion and macrophage chemotaxis [104].

Some ferroptosis inhibitors also ameliorate fibrosis. Both ferroptosis and fibrosis were ameliorated after administration of ferroptosis inhibitor liproxstatin-1 in radiation-induced lung fibrosis [126] in an IRI model [104] and a unilateral ureteral obstruction (UUO) model [103]. In addition to ferroptosis inhibitors, other drugs can exert both antiferroptotic and anti-fibrotic effects. Nrf2 is a transcription factor that regulates the activity of many genes involved in iron metabolism [127]. FG-4592 plays a protective role in folic acid-induced renal injury and delays the progression of renal fibrosis by activating Nrf2 to inhibtit ferroptosis [101]. Tocilizumab and small-molecule drugs from traditional Chinese medicine ingredients such as nobiletin and tectorigenin have all been shown to inhibit fibrosis and ferroptosis progression [128–130].

Considering safety and other concerns, research on these medications is currently limited to the experimental stage, and their clinical application is still restricted. Targeting the site of injury without disrupting normal metabolic pathways remains a challenge. Nonetheless, understanding ferroptosis and AKI in CKD is benefit in exploring new therapeutic approaches.

## Outlook

In this review, we have explored the fundamentals of ferroptosis and how it governs interstitial fibrosis, mitochondrial dysfunction, inflammatory responses, tubular cell regeneration, and other cellular processes in the progression of AKI to CKD. Finally, we briefly describe the crosstalk between these mechanisms. We concluded that ferroptosis might be a driver in converting renal maladaptation to CKD and was a promising therapeutic target to halt disease progression.

Still and all, several questions need to be addressed in the future. The efficacy of ferroptosis inhibitors is yet to be blank in clinical settings. Although compounds like ferrostatin-1 and liproxstatin-1 have shown positive results in animal models, further studies are necessary to ensure their safety and feasibility for human use. It’s regrettable that research on some clinically used drugs is still confined to animal models, althought their characteristics well documented. For example, recent studies showed that paricalcitol inhibits cisplatin-induced AKI by activating the vitamin D receptor to regulate the antioxidant effect of GPX4 [131]. Iron chelators deferoxamine could alleviate ferroptosis and fibrosis in CKD rats [132]. Antioxidants, like vitamin E and melatonin, face similar challenges. Notably, there are currently no particular markers to identify ferroptosis in vivo, and numerous other kinds of cell death are implicated in the pathophysiology of kidney injury. Therefore, direct evidence of ferroptosis attenuation in vivo after drug treatment is still lacking, and more in-depth studies are needed [133]. The mechanisms involved in ferroptosis also need to be further explored. In addition to ferroptosis occurring in the tubules themselves, it has been shown that glomerular injury in patients with lupus nephritis triggers ferroptosis in tubules [134], therefore we hypothesized that the interaction between glomeruli and renal tubules plays an important role in the progression from AKI to CKD, and we await further studies on this topic with much anticipation.

Despite the concerns, we strongly believe that investigating the role of ferroptosis in kidney injury is vital and worthwhile. We anticipate that large-scale studies in this field will help us to comprehend the pathogenesis and heterogeneity of AKI to CKD and provide the groundwork for preventing disease progression and identifying viable treatment targets.

## Data Availability

The data of this study are included within the paper.
